# Bypass Pyeloplasty for Ureteropelvic Junction Obstruction Associated with Horseshoe Kidney: A Case Report

**DOI:** 10.70352/scrj.cr.25-0729

**Published:** 2026-04-21

**Authors:** Tsubasa Shironomae, Keiko Ainoya, Kiyohide Sakai

**Affiliations:** Department of Urology, Miyagi Children’s Hospital, Sendai, Miyagi, Japan

**Keywords:** bypass pyeloplasty, high ureteral insertion, horseshoe kidney, hydronephrosis, ureteropelvic junction obstruction

## Abstract

**INTRODUCTION:**

Horseshoe kidney (HSK) is the most common renal fusion and malrotation anomaly, and ureteropelvic junction obstruction (UPJO) due to high ureteral insertion (HUI) is frequently associated with HSK. Bypass pyeloplasty (BP) is a non-dismembered surgical technique involving a side-to-side anastomosis between the ureter just distal to the UPJO and the dependent portion of the hydronephrotic renal pelvis, and is suited for HUI. Although several procedures have been suggested for UPJO in HSK patients, BP has not been discussed much. In this case report, we describe the detailed procedure for BP successfully performed in an HSK patient with UPJO due to HUI.

**CASE PRESENTATION:**

The patient was a girl aged 1 year and 10 months who had experienced intermittent abdominal pain and occasional vomiting for 1 month. She was diagnosed with dilation of the left renal pelvis and renal calyces (Society for Fetal Urology classification grade 3–4) on ultrasonography and subsequently referred to our hospital for diagnosis of left intermittent hydronephrosis. Renal scintigraphy revealed the HSK, and the differential renal function (DRF) in the left kidney was 16.3%. At 1 year and 11 months, she underwent retrograde left ureteropyelography and pyeloplasty. The retrograde left ureteropyelography showed the HUI and obstruction of the left ureter at approximately 45 mm from the cephalic edge of the umbilicus. We performed a 3.0-cm left flank incision to expose the left renal pelvis and ureter through the retroperitoneal route. HUI of the left ureter and crossing of a white cord-like structure that compressed the left ureter and renal pelvis just distal to the UPJO were identified. We divided the white cord-like structure and performed BP between the ureter just distal to the UPJO and the dependent portion of the renal pelvis. The postoperative course was good, with improvement in the renal pelvis dilation and the DRF in the left kidney. There has been no recurrence for >6 years after surgery.

**CONCLUSIONS:**

We recommend BP as a physiological and suitable surgical approach that reduces the risk of impaired blood supply and postoperative anastomotic stenosis in HSK patients with UPJO due to HUI.

## Abbreviations

BP
: bypass pyeloplasty
DRF
: differential renal function
HSK
: horseshoe kidney
HUI
: high ureteral insertion
LP
: laparoscopic pyeloplasty
OP
: open pyeloplasty
RALP
: robot-assisted laparoscopic pyeloplasty
SFU
: Society for Fetal Urology
UPJO
: ureteropelvic junction obstruction

## INTRODUCTION

HSK is the most common renal fusion and malrotation anomaly, occurring in approximately 1 in 500 births.^1)^ UPJO is frequently associated with HSK, being observed in 15%–33% of HSK patients.^2)^ Most HSK patients are asymptomatic, and a surgical intervention is not required unless a complicated UPJO is present.^2,3)^ The high incidence rate of UPJO in HSK patients arises through various unusual anatomies,^2,4)^ and several surgical methods have been proposed to date.

BP is a non-dismembered surgical technique involving a side-to-side anastomosis between the ureter just distal to the UPJO and the dependent portion of the hydronephrotic renal pelvis, and is ideally suited for HUI.^5)^ In this case report, we describe the detailed procedure for BP successfully performed in an HSK patient with UPJO due to HUI.

## CASE PRESENTATION

**Fig. 1:**
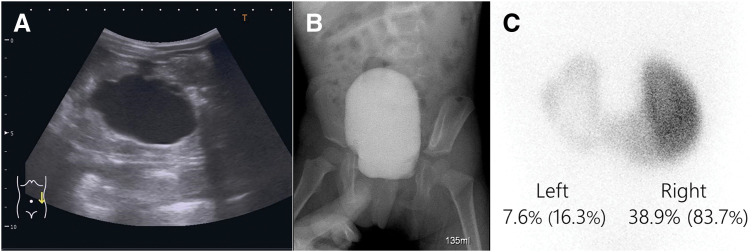
Preoperative examination images. (**A**) Ultrasonography showed dilation of the left renal pelvis and renal calyces (SFU classification grade 3–4). (**B**) Voiding cystourethrography showed no vesicoureteral reflux. (**C**) Renal scintigraphy revealed the HSK, and the DRF in the left kidney was 16.3%. DRF, differential renal function; HSK, horseshoe kidney; SFU, Society for Fetal Urology

The patient was a girl aged 1 year and 10 months who had experienced intermittent abdominal pain and occasional vomiting for 1 month. She was diagnosed with dilation of the left renal pelvis and renal calyces (SFU classification grade 3–4) on ultrasonography (**Fig. 1A**), and subsequently referred to our hospital by a pediatrician for diagnosis of left intermittent hydronephrosis. Vesicoureteral reflux was not observed on voiding cystourethrography (**Fig. 1B**). Renal scintigraphy using technetium-99m dimercaptosuccinic acid revealed the HSK, and the DRF in the left kidney was 16.3% (**Fig. 1C**).

**Fig. 2:**
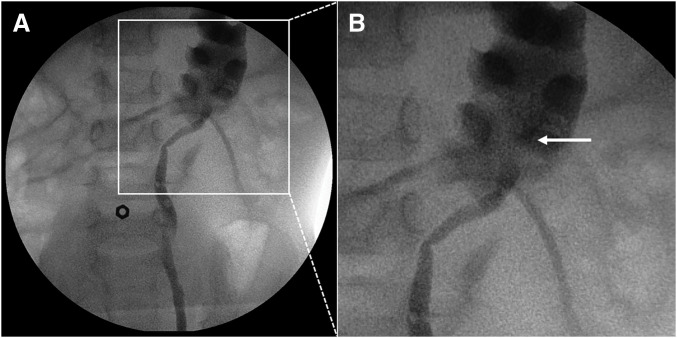
Retrograde left ureteropyelography images. (**A**, **B**) Retrograde left ureteropyelography showed high insertion of the left ureter (white arrow) and obstruction of the left ureter at approximately 45 mm from the cephalic edge of the umbilicus. The hexagonal metal ring shows the position of the umbilicus.

**Fig. 3:**
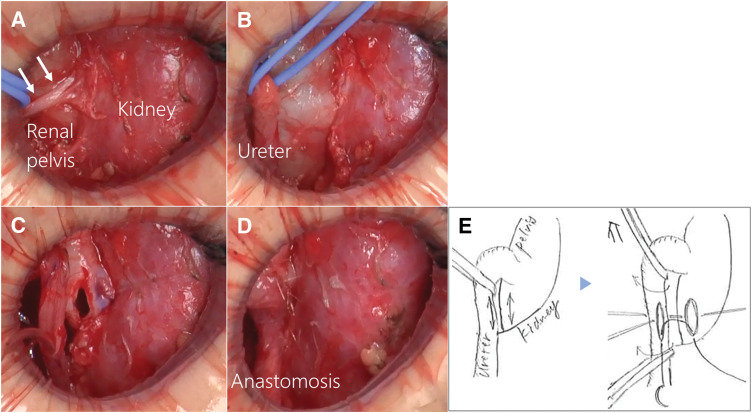
Intraoperative findings. (**A**) The crossing of the white cord-like structure (white arrows) that compressed the left ureter and renal pelvis just distal to the UPJO was ligated and divided. (**B**–**D**) BP between the ureter just distal to the UPJO and the dependent portion of the renal pelvis was performed. (**E**) Schematic illustrations of the intraoperative findings in (**B**) and (**C**). BP, bypass pyeloplasty; UPJO, ureteropelvic junction obstruction

At 1 year and 11 months, she underwent retrograde left ureteropyelography and pyeloplasty. The retrograde left ureteropyelography showed the HUI and obstruction of the left ureter at approximately 45 mm from the cephalic edge of the umbilicus (**Fig. 2**). We performed a 3.0-cm left flank incision off the tip of the 11th rib to expose the left renal pelvis and ureter through the retroperitoneal route. HUI of the left ureter and crossing of a white cord-like structure that compressed the left ureter and renal pelvis just distal to the UPJO were identified (**Fig. 3A**). The white cord-like structure was ligated and divided. We concluded that the successful performance of dismembered pyeloplasty was not feasible because the left renal pelvis was not adequately dilated and the left renal parenchyma and isthmus were located near the left renal pelvis. Furthermore, the isthmus did not appear to be the cause of the ureteral obstruction. Therefore, we performed BP, involving a 12-mm side-to-side anastomosis between the ureter just distal to the UPJO and the dependent portion of the renal pelvis (**Fig. 3B**–**3E**). The anastomosis was completed with 6-0 Monocryl (poliglecaprone 25) on the posterior wall in a running manner and on the anterior wall in an interrupted manner. A 3.7-Fr double-J ureteral stent was inserted anterogradely before the anastomosis of the anterior wall, with no drain in the retroperitoneal space. The operation time was 139 min.

**Fig. 4:**
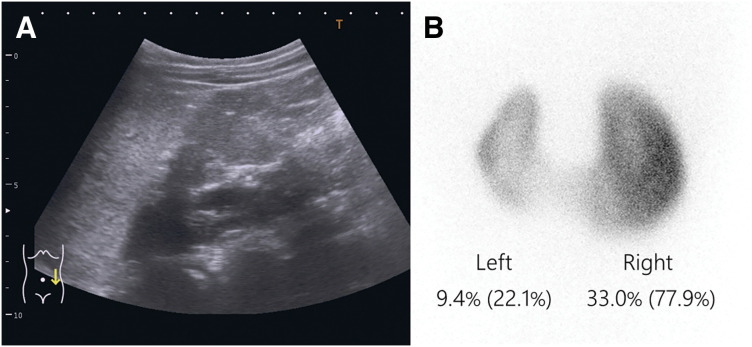
Postoperative examination images. (**A**) Ultrasonography showed mild dilation of the left renal pelvis (SFU classification grade 1). (**B**) Renal scintigraphy revealed that the DRF in the left kidney was 22.1%. DRF, differential renal function; SFU, Society for Fetal Urology

The postoperative course was good. The urethral catheter was removed on POD 1, and the patient was discharged on POD 3. The double-J ureteral stent was removed on POD 48. Mild dilation of the left renal pelvis (SFU classification grade 1) was detected on ultrasonography (**Fig. 4A**), and the DRF in the left kidney was 22.1% on renal scintigraphy (**Fig. 4B**) in postoperative year 6. Both findings indicated improvement from the preoperative results.

## DISCUSSION

BP involves a side-to-side anastomosis between the ureter and the hydronephrotic renal pelvis and is ideally suited for UPJO due to HUI.^5)^ The rationale for this approach lies in the fact that the UPJO is partial and preserving the ureteropelvic junction can minimize potential injury to the blood supply, and that it is helpful to add another outlet via a bypass procedure.^5)^ HUI was observed in 20%–80% of HSK patients with UPJO in a previous study.^2)^ Although several procedures have been suggested for HSK patients with UPJO to date,^2–4,6)^ BP has not been discussed much. Furthermore, hydronephrosis in HSK patients does not necessarily require correction, but it does require careful evaluation for all possible causes of obstruction.^4)^

Minimally invasive surgery for HSK patients with UPJO is an important issue. OP for HSK patients with UPJO is considered the gold standard modality and has a high success rate.^2)^ Culp et al.^4)^ have been the only authors to report BP-like procedure outcomes for HSK patients with UPJO, with 5 of their 6 patients exhibiting good postoperative courses. Although Pitts et al.^3)^ demonstrated that Foley Y-V pyeloplasty, another non-dismembered surgical technique, was highly effective for HSK patients with UPJO, the procedure is associated with a risk of postoperative anastomotic stenosis. While long anastomotic times and intracorporeal knot-tying difficulties are the main obstacles during LP, the introduction of RALP has solved these problems.^2)^ Both LP and RALP basically require a transperitoneal approach to ensure a wide field of view, but there is a risk of damage to the intra-abdominal organs. Because the high incidence rate of UPJO in HSK patients arises through various unusual anatomies,^2,4)^ the usefulness of OP may be universal.

In the present case, we concluded that conventional dismembered pyeloplasty was not feasible because the left renal pelvis was not adequately dilated and the left renal parenchyma and isthmus were located near the left renal pelvis. Based on the above background, we selected BP for this HSK patient with UPJO due to HUI and safely performed the procedure in a shallow surgical field using surgical loupes. The postoperative course was good, with improvement in the renal pelvis dilation and the DRF in the left kidney, and no recurrence for >6 years after surgery. Thus, for patients with UPJO due to HUI with HSK, BP appears to be a physiological and suitable surgical approach that can reduce the risk of impaired blood supply and postoperative anastomotic stenosis. When the anastomosis is performed during BP, the use of appropriate tension by supporting sutures is important for a meticulous operation. It is also important to use a direction that is as unaffected as possible when the ureter passes through the ventral side of the isthmus. Although non-dismembered pyeloplasty involving BP cannot detect UPJO pathologically, it is considered an appropriate surgical procedure that can surpass this issue.

## CONCLUSIONS

We performed BP in an HSK patient with UPJO due to HUI, and the postoperative course was good. Based on our experience, we recommend BP as a physiological and suitable surgical approach that reduces the risk of impaired blood supply and postoperative anastomotic stenosis in patients with UPJO due to HUI with HSK.

## DECLARATIONS

### Availability of data and materials

The data that support the findings of this study are available from the corresponding author, Tsubasa Shironomae, upon reasonable request.
